# A complete, multi-layered quranic treebank dataset with hybrid syntactic annotations for classical arabic processing

**DOI:** 10.1016/j.dib.2025.111940

**Published:** 2025-08-05

**Authors:** Wadee A. Nashir, Abdulqader M. Mohsen, Asma A. Al-Shargabi, Mohamed K. Nour, Badriyya B. Al-onazi

**Affiliations:** a Department of Computer Science, Faculty of Computing and Information Technology, University of Science and Technology, Yemen; b Faculty of Computer and Information Technology, University of Aden, Aden, Yemen; c Department of Information Technology, Collage of Computer, Qassim University, Buraydah 51452, Saudi Arabia; d Department of Computer Sciences, College of Computing and Information System, Umm Al-Qura University, Saudi Arabia; e Department of Language Preparation, Arabic Language Teaching Institute, Princess Nourah bint Abdulrahman University, P.O. Box 84428, Riyadh 11671, Saudi Arabia

**Keywords:** Holy quran, Treebank, Syntactic analysis, Morphological analysis, Linguistic resources, Dependency Parsing, Constituency Parsing

## Abstract

This article describes the Extended Quranic Treebank (EQTB), a comprehensive, multi-layered, and computationally accessible linguistic resource for Classical Arabic (CA), meticulously developed to overcome the documented limitations of the original Quranic Treebank. Leveraging foundational data from established Quranic digital resources, EQTB features systematically expanded orthographic representations generated via algorithmic processing and validation; rigorously refined morphological annotations based on expanded expert-informed schemas, automated re-annotation, and manual curation; and critically, a novel, complete syntactic layer constructed through algorithmic conversion of prior graphical data, Deep Learning-based parsing achieving full coverage under a hybrid constituency-dependency framework, and expert validation. Encompassing the entire Quran (∼132,736 tokens), the dataset is structured in an adapted CoNLL-X format across 43 columns, detailing multiple orthographies, fine-grained morphology (45 tags), and complete hybrid syntax (140 tags/labels), complemented by auxiliary lexicons and schemas. EQTB offers significant reuse potential, providing crucial training/evaluation data for diverse CA NLP tasks (parsing, morphology, diacritization), supporting linguistic research, and enabling the development of advanced pedagogical tools and language technologies.

Specifications Table

**Table:** 

Subject	*Computer Science*
Specific subject area	Artificial Intelligence (Natural Language Processing NLP)
Type of data	Table; Processed; Analyzed. (Added Types): Annotated Data; Lexical Resource Data Structured Tables (CoNLL-X format) containing processed, analyzed, annotated, and curated linguistic data (orthographic, morphological, syntactic layers). Includes auxiliary lexical resource Tables (text format).
Data collection	Base Quranic text data (Uthmani and Imlaai scripts) were sourced from publicly available Tanzil and Quranic Corpus resources. Custom Python scripts were developed and employed for multi-level tokenization, Buckwalter/phonetic transliteration generation, and English translation alignment. Morphological annotations were programmatically re-assigned based on refined, expert-informed schemas derived from authoritative linguistic references. Syntactic analysis seed data (parse tree images) were programmatically collected (web-crawled) from the Quranic Corpus, then converted algorithmically (Python) into a structured format to subsequently train a Deep Learning parser responsible for generating annotations across the entire corpus.
Data source location	Initial data were sourced from public digital resources (Tanzil Project [1], Quranic Corpus [2], Comprehensive Islamic Library [3]). Subsequent dataset development and curation were conducted within academic research settings in Department of Computer Science, Faculty of Computing and Information Technology, University of Science and Technology, Yemen.
Data accessibility	**Repository name:** Mendeley Data **Data identification number:** doi: 10.17632/rk96pn66m4.1 **Direct URL to data:**https://data.mendeley.com/datasets/rk96pn66m4/1 **Instructions for accessing these data:** The dataset ('Quranic') is publicly hosted on Mendeley Data. Access is provided via the DOI link above. Users can directly navigate the URL to view dataset details and download associated files (primary data in extended CoNLL-X format and auxiliary resources). No registration or special permissions are required for access.

## 1 Value of the Data

• This dataset represents a landmark contribution to Classical Arabic (CA) computational linguistics. It provides the first complete, computationally accessible, and meticulously annotated treebank for the entire Quran, addressing the critical limitations of its predecessor—which offers only partial (∼40 %) and largely non-processable syntactic annotations. Engineered for robust processing under a hybrid Constituency-Dependency framework, its multi-layered scheme offers unparalleled depth: it furnishes dual Uthmani and Imlaai orthographies for broader applicability, features exceptionally granular morphological analysis, and critically, introduces a novel syntactic layer that explicitly models complex CA phenomena like ellipsis. By doing so, it establishes a new and essential benchmark for the development, training, and rigorous evaluation of sophisticated NLP models, moving far beyond existing resources in scope, depth, and usability.
• Researchers can directly reuse this dataset to advance various frontiers in CA processing and historical linguistics. The granular, interlinked annotations provide high-quality training and testing data for tasks such as automatic diacritization of classical texts, morphological analysis and disambiguation specific to CA, and robust Constituency Parsing [4]. Furthermore, it enables novel explorations into the intricate relationship between morphology and syntax within the historical context of CA, offering insights valuable for both computational modelling and linguistic theory.
• The treebank possesses substantial pedagogical utility for advanced studies in CA linguistics, Arabic philology, and Quranic exegesis (Tafseer). Its structured data, illustrating orthographic conventions, complex morphological derivations, and syntactic constructions directly from the Quran, serves as an invaluable resource for curriculum development and in-depth learning [5]. It facilitates a nuanced understanding of CA grammar and aids students, including non-native speakers, in mastering the linguistic intricacies of the Quranic text and other classical literature.
• This dataset provides a readily deployable foundation for creating sophisticated digital tools and applications centred on CA and the Holy Quran, mitigating the significant effort required for de novo resource creation. Developers and researchers can utilize this structured data to build advanced applications such as specialized semantic search engines for classical texts, enhanced digital Tafseer platforms integrating linguistic analysis, dedicated Quranic computational linguistic tools, and innovative CA learning software incorporating modern pedagogical approaches (e.g., visualization, adaptive learning) grounded in authentic linguistic examples.
• By providing a substantial, computationally accessible resource for the historically under-resourced domain of CA, this treebank significantly lowers the barrier for entry into CA NLP research. It furnishes a standardized dataset enabling robust cross-linguistic structural comparisons with other Semitic languages or Modern Standard Arabic, facilitating empirical investigations in linguistic typology and historical linguistics cantered on authentic CA data.
• The detailed, multi-layered annotations, coupled with the hybrid grammatical framework grounded in the stable Quranic text, provide rich empirical data for investigating and evaluating specific theoretical linguistic hypotheses concerning CA syntax, morphology, and their interface. Furthermore, the public availability and consistent structure of this dataset can foster methodological convergence and promote the development of standardized annotation practices within the CA linguistic and computational research communities [6].

## 2 Background

The advancement of Natural Language Processing (NLP) for CA is significantly hindered by the scarcity of comprehensive, computationally tractable linguistic resources. The primary existing resource, the Quranic Arabic Dependency Treebank (QADB), while valuable, exhibits critical limitations: its orthography employs Quran-specific conventions unsuitable for general CA texts; its morphological annotation lacks completeness; and its syntactic layer offers limited coverage (approximately 40 %) and is primarily available in non-processable image formats, thus failing to represent CA's grammatical complexity adequately for computational use [7]. The principal motivation for developing this dataset was to address this critical resource gap by creating the first comprehensive, multi-layered (orthographic, morphological, syntactic) CA Treebank derived from the entire Quran, specifically engineered for computational accessibility and usability. The methodology involved algorithmic reconstruction and enhancement of the orthographic and morphological layers, development of a complete syntactic parsing model using Deep Learning, and a rigorous revision and expansion of the morphological and syntactic tagsets grounded in authoritative linguistic references from the classical Arabic grammatical tradition.

## 3 Data Description

This dataset presents a significantly expanded and computationally enhanced CA Treebank, derived from the entirety of the Holy Quran. It builds upon and systematically supersedes the QADB [2,7], addressing its documented limitations through substantial extensions in both scope (vertical expansion to cover all verses) and detail (horizontal expansion via numerous additional annotation columns). The resource is primarily organized into a central, comprehensive data file, designated Quranic, which is complemented by several auxiliary files containing lexical information and annotation scheme definitions.

**Fig. 1:**
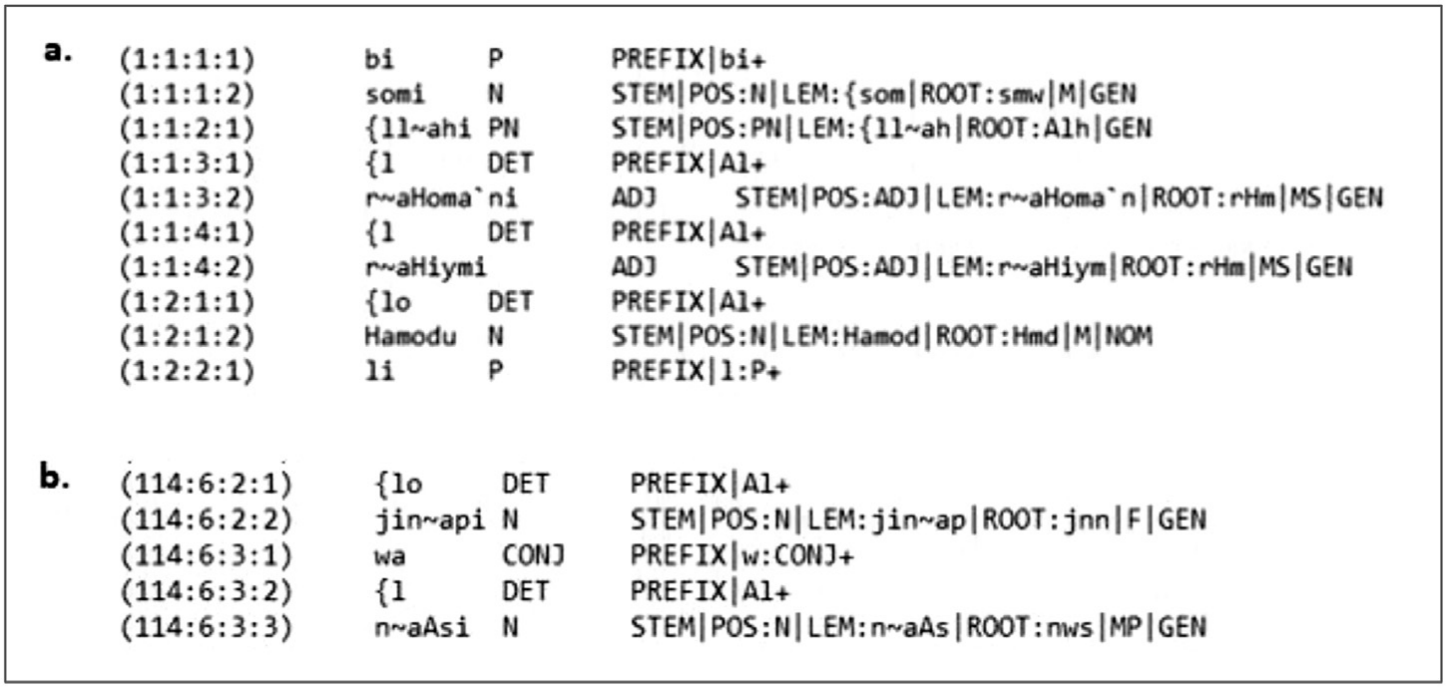
A snapshot of the primary table of original corpus QADB. (a) The first 10 rows of QADB. (b) The 5 last rows of QADB.

The Quranic file meticulously integrates three linguistic layers—orthographic, morphological, and syntactic—for the complete Quranic text, comprising approximately 140,000 tokens. Data is structured according to an adapted CoNLL-X format [8]. The CoNLL-X format is a widely adopted standard in computational linguistics for distributing annotated linguistic data, particularly in dependency parsing. It is a tab-separated, text-based format in which each line corresponds to a single token, and each column represents a distinct linguistic feature, such as the token’s index, surface form, lemma, part-of-speech (POS), and syntactic dependencies. One of the strengths of the CoNLL-X format lies in its flexibility—it can be readily extended to include additional columns and linguistic features, which makes it well-suited for representing languages with complex morphological and syntactic structures, such as CA. This dataset builds on that flexibility by incorporating numerous additional fields to capture the unique characteristics of CA. This represents a stark contrast to the original QADB [2] (illustrated partially in Fig. 1), which contained only four primary columns (token location, Uthmani script token, POS, basic morphological features) and lacked a complete, computationally processable syntactic layer.

The enhanced Quranic table in this dataset encompasses a total of 43 columns, providing fine-grained linguistic detail. While 36 columns are dedicated to enriched orthographic and morphological representations—extracted, processed, and refined from the original QADB data to improve usability and analytic potential—the principal contribution lies in the 7 entirely new columns constituting a complete syntactic annotation layer for the entire Quran. This novel layer employs expanded tagsets (140 syntactic tags/labels). The subsequent subsections provide a detailed breakdown of the data structure within each of the three linguistic layers.

### 3.1 Orthographical layer data

The orthographical layer within the primary Quranic data table significantly enhances the representation provided in the original QADB by furnishing multiple perspectives on each token. Moving beyond the previous limitation of only Uthmani script (specific to Quranic texts) and a Quran-centric positional code, this expanded dataset incorporates crucial additions for broader utility. Critically, it includes a parallel representation of each token in the Imlaai script, the standard orthography for general CA texts, thereby greatly improving the dataset's applicability for models intended for wider CA corpora. Furthermore, Buckwalter transliteration is provided, ensuring seamless interoperability with numerous existing Arabic NLP resources and tools reliant on this widely adopted ASCII standard. To bolster pedagogical applications and accessibility for non-native speakers engaging with CA, columns containing English transliteration and English translation are also integrated. Addressing usability challenges posed by the Quran's unique structure, the dataset implements a dual indexing system: alongside the traditional hierarchical Quranic location code (chapter, verse, word, token), a linear sentence-based coding is introduced, rendering the data more compatible with standard NLP processing pipelines. Table 1 offers a detailed specification of each column within this enriched orthographic layer, which collectively provides essential data for research in CA tokenization, automatic diacritization, and the development of educational resources.

**Table 1:** Summary of Orthographical Layer Columns in the Primary Quranic Table.

This meticulously curated, multi-representational orthographic layer provides a significantly enhanced foundation for diverse computational linguistics research and advanced pedagogical applications targeting CA. Specifically, the inclusion of the standard Imlaai script (imlaai_token, imlaai_unicode) alongside the specialized Uthmani form (uthmani_token) enables the development and evaluation of NLP models (e.g., for tokenization, automatic diacritization, morphological analysis) possessing broader generalizability across various CA texts beyond the Quran. Concurrently, the provision of Buckwalter transliteration (uthmani_unicode) facilitates crucial interoperability, allowing seamless integration with numerous existing Arabic digital resources, lexicons, and established NLP pipelines. The implementation of a dual indexing system (sentence-based sentence_id, sentence_word, token_id and Quran-based location,chapter_ id, verse_id, word_id, tok_id) offers critical flexibility, empowering researchers to adopt analytical frameworks suited either to standard sentence processing or to the unique structural properties of the Quran. Furthermore, the integrated phonetic transliteration (phonetic) and English translation (trans) substantially augment the dataset's value, serving as vital input for creating sophisticated language learning materials, enhancing text comprehension for learners, particularly non-native speakers, and opening potential avenues for cross-lingual alignment and comparative linguistic studies [9].

### 3.2 Morphological layer data

Addressing the profound morphological complexity characteristic of CA and overcoming the limitations of the original QADB, this layer presents a deeply granular and systematically expanded analysis. This expansion was guided by a meticulous redesign of the annotation schema which, as will be detailed in the *Morphological Layer Enhancement* section, was developed to harmonize traditional grammatical analyses with the requirements of computational processing. Contained within the primary Quranic data file are 21 dedicated columns for morphology (a stark contrast to the original two), reflecting meticulous data cleaning and significant enrichment of annotations, as detailed comprehensively in Table 2. This enhanced layer provides multi-faceted lexical insights, including distinct representations for lemma and root in both Arabic script and standard transliteration/Unicode, alongside precise token segmentation identifying constituent prefixes, stems, and suffixes.

**Table 2:** Summary of Morphological Layer Columns in the Primary Quranic Table.

Crucially, the annotation extends to a highly refined Part-of-Speech (POS) tagging system, offering perspectives aligned with both computational conventions and the nuances of traditional CA grammar. Beyond lexical and POS information, the dataset encodes an extensive suite of fine-grained morphosyntactic features critical for linguistic analysis. These encompass detailed specifications for verbal properties (form/pattern, aspect, mood, voice, person), nominal attributes (case, state/definiteness, gender, number), types of derived nouns, and the identification of words belonging to special grammatical classes (e.g., *kāna wa-akhawātuhā*).

To ensure clarity and facilitate accurate interpretation and utilization of these rich annotations, the dataset is accompanied by essential auxiliary resources, summarized in Table 3. These include comprehensive, bilingual (Arabic/English) root and lemma lexicons (CARootLexicon, CALemmaLexicon) derived from the corpus, alongside meticulously defined schemas specifying the complete POS tagset (CAPoS) and the full inventory of morphological features employed (CAMorphFeatures). This systematically organized, deeply annotated, and clearly documented morphological layer furnishes an unprecedented foundation for advancing CA Natural Language Processing tasks—such as morphological analysis, disambiguation, lemmatization, and POS tagging—while also providing invaluable empirical data for sophisticated computational modeling of the morphosyntax interface and supporting rigorous theoretical linguistic investigations into CA structure.

**Table 3:** Summary of Auxiliary Files for the Morphological Layer.

File name	Content Description	Key Statistics/Scope
CARootLexicon	Lexicon providing unique CA roots identified within the corpus.	1642 unique roots derived from 77,439 words.
CALemmaLexicon	Lexicon providing unique CA lemmas identified within the corpus.	4832 unique lemmas derived from 77,439 words.
CAPoS	Specification and definition of the Part-of-Speech (POS) tagset employed.	Defines 45 primary POS tags (further detailed by features).
CAMorphFeatures	Comprehensive schema defining all morphological features (including those in Table 2).	Detailed specifications for nominal, verbal, particle features.

## 4 Syntactic Layer Data

Constituting the pivotal contribution of this dataset, the syntactic layer offers entirely new, comprehensive annotations covering the full Quranic text, addressing a critical gap in existing CA resources. Recognizing the inherent complexity of CA syntax, which often necessitates a blend of dependency and constituency perspectives [7], this layer is meticulously engineered based on an extended CoNLL-X format [8]. The annotation scheme itself was systematically developed from authoritative linguistic sources to ensure that the captured data is both computationally tractable and grammatically robust, as will be detailed in the *Syntactic Layer Building* section. This adaptation specifically accommodates a hybrid constituency-dependency representation and includes mechanisms for handling elliptical constructions. The structure comprises 7 dedicated columns within the primary Quranic table, designed to capture this rich syntactic information, as specified in Table 4.

**Table 4:** Summary of Syntactic Layer Columns in the Primary Quranic Table.

**Fig. 2:**
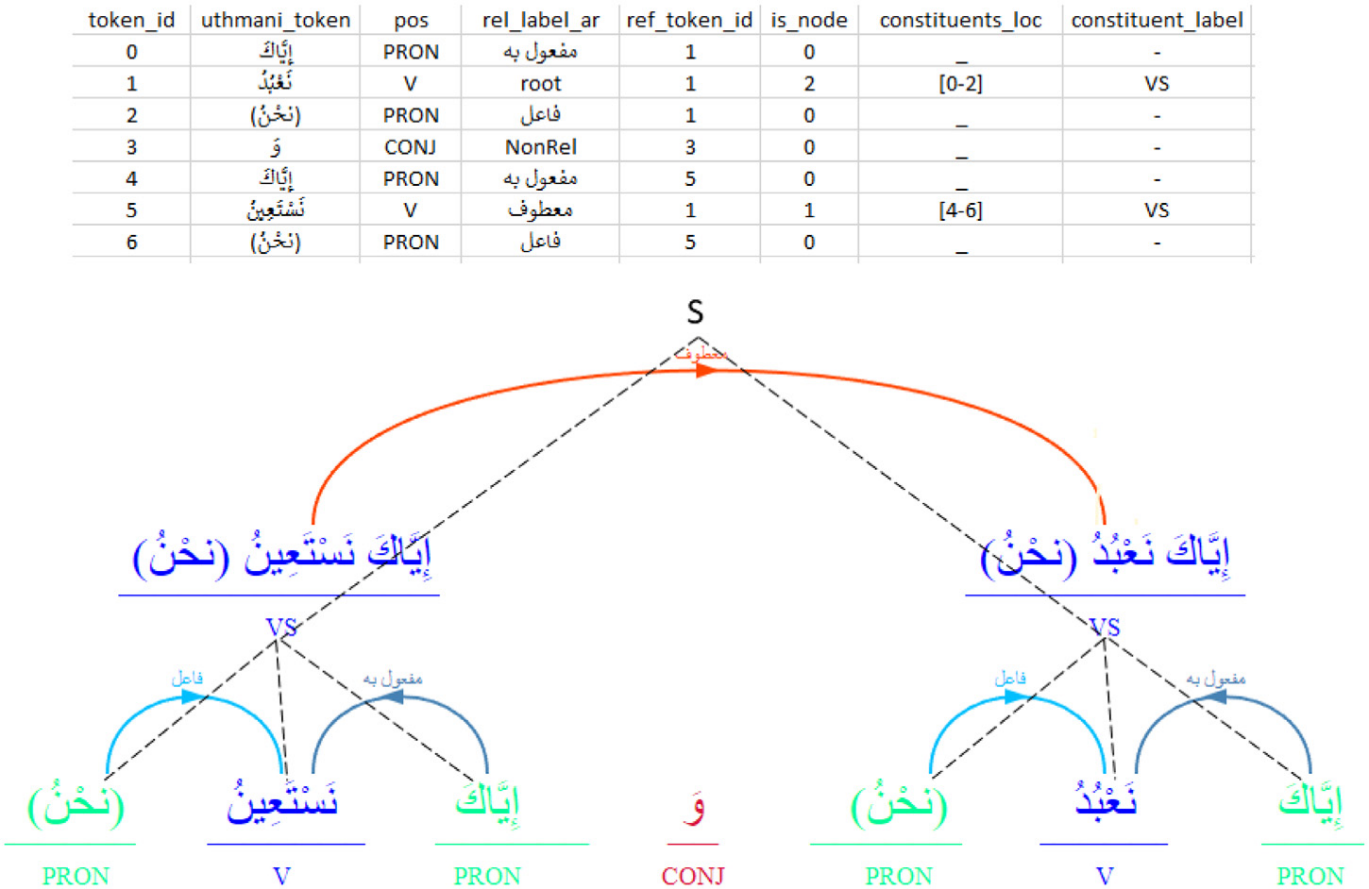
Data for Hybrid Constituency-Dependency Representations.

These columns provide integrated details for both grammatical paradigms. Dependency relations are captured through explicit identification of head tokens (ref_token_id) and assigned relation labels, provided in both standard English terminology (rel_label) and nomenclature derived from traditional Arabic grammar (rel_label_ar). Notably, the annotation scheme explicitly marks tokens lacking a syntactic head (Non_Head). Concurrently, constituency information is encoded via flags identifying constituent heads (is_constituent), columns defining the exact span (start/end tokens) (constituents_loc) and surface form (constituents) of headed phrases, and labels specifying the syntactic category of these constituents (constituent_label) (e.g., Nominal Sentence, Verbal Sentence, Prepositional Phrase). Fig. 2 provides a visual example illustrating how this hybrid representation is captured in the data.

To adequately model CA's syntactic intricacies, the annotation employs highly fine-grained schemas. The set of dependency relation labels has been significantly expanded, encompassing 140 distinct binary relations defined in the RelLabels auxiliary file. Similarly, the types of phrasal constituents are defined in the ConstituentsTags auxiliary file. Descriptions for these crucial auxiliary resources are summarized in Table 5. This novel, computationally accessible syntactic layer provides an unprecedented and robust foundation for developing and evaluating CA parsers (dependency, constituency, or hybrid), facilitating empirical testing of syntactic theories, enabling comparative grammatical studies, and supporting the creation of advanced syntax-aware CA language technologies.

**Table 5:** Summary of Auxiliary Files for the Syntactic Layer.

Filename	Content Description	Key Statistics/Scope
RelLabels	Defines the comprehensive set of 140 distinct syntactic dependency relation labels used.	140 unique relation labels.
ConstituentsTags	Defines the set of 7 distinct phrasal constituent category tags used in the analysis.	7 unique constituent tags.

To fully contextualize the novelty and significance of this syntactic layer, a direct comparison with existing Arabic treebanks is essential. Prominent resources like the Penn Arabic Treebank (PATB) [10], Prague Arabic Dependency Treebank (PADT) [11], and Columbia Arabic Treebank (CATB) have been foundational for Modern Standard Arabic (MSA) [12] but are less suited for Classical Arabic (CA) due to differences in domain and their use of grammatical frameworks not originally designed for CA's unique structures.

The most relevant predecessor, the original Quranic QADB [2], was a pioneering effort but presented critical barriers to computational use. Most notably, its syntactic layer is incomplete, covering only ∼40 % of the Quran, and is available primarily as non-processable static images. This renders it unusable for training or evaluating modern NLP models. In contrast, the EQTB provides the first complete (100 % coverage) and fully machine-readable (CoNLL-X format) syntactic annotation for the entire Quranic text. Beyond full coverage, EQTB introduces several critical enhancements previously unavailable in any resource, such as the explicit annotation of elliptical constructions, the use of traditional Iʿrāb terminology, and a dual indexing system designed for both linguistic research and NLP applications. Furthermore, its hybrid constituency-dependency framework, grounded in traditional Arabic grammar, offers a more faithful representation of CA syntax than other models. The following table highlights these crucial distinctions Table 6.

**Table 6:** Comprehensive Comparison of Major Arabic Treebanks.

Criteria	PATB	PADT	CATiB	QADT (Original)	EQTB (This Work)
Domain & Language	MSA (Newswire)	MSA (Newswire)	MSA(Multi-domain)	CA (Quran)	CA (Quran)
Grammatical Framework	Constituency (English-style)	Dependency (Czech-style)	Simplified Dependency	Hybrid (Traditional Arabic Grammar)	Hybrid (Constituency-Dependency) grounded in Traditional Arabic Grammar
Syntactic Coverage	Full	Full	Full	Partial (∼40 %)	**Complete** (100 % of the Quran)
Computational Accessibility (Syntactic)	High (Text format)	High (Text format)	High (Text format)	Very Low (Primarily non-processable images)	**Very High** (Fully machine-readable CoNLL-X text format)
Syntactic Annotation Depth	Standard constituency labels	Standard dependency labels	Simplified (8 dependency relations)	Fine-grained (inaccessible)	**Very High** (140 dependency labels, ellipsis handling)
Morphological Depth	High (Extensive tagset)	Detailed (Positional encoding)	Shallow (6 POS tags)	Very High (Comprehensive feature set)	**Extremely High** (21 feature columns)
Script Diversity	Imlaai only	Imlaai only	Imlaai only	Uthmani only	Uthmani + Imlaai (Parallel representation)
Indexing System	Hierarchical (for coreference & traces)	Sequential	Sequential	Hierarchical (Sura:Verse)	**Dual**: Hierarchical (Quranic) and Linear (Sentence-based)
Inter-Annotator Agreement	Reported	Reported	Reported	Not Reported	**Not Computed** (Validation via expert protocol against 7 classical references)
Tool & Infrastructure Support	LDC tools	Various	Limited	High (Web interface, downloadable data)	High: Open data (Mendeley), Lexicons, Schemas, Public visualization tool
Classical Grammatical Terminology (Syntactic)	No	No	No	Implicit (in images)	**Yes** (Dedicated column rel_label_ar for Iʿrāb labels)
Ellipsis & Reconstruction Annotation	Yes (Explicit placeholder for ellipsis)	Yes (At tectogrammatical level)	No	Implicit (in images)	**Explicit** (Systematically handled via TAQDIR module and annotation)
Sentence-Level Segmentation for NLP	Yes (by default)	Yes (by default)	Yes (by default)	No (Verse-based structure)	**Yes** (Dedicated linear sentence IDs alongside Quranic structure)

**Fig. 3:**
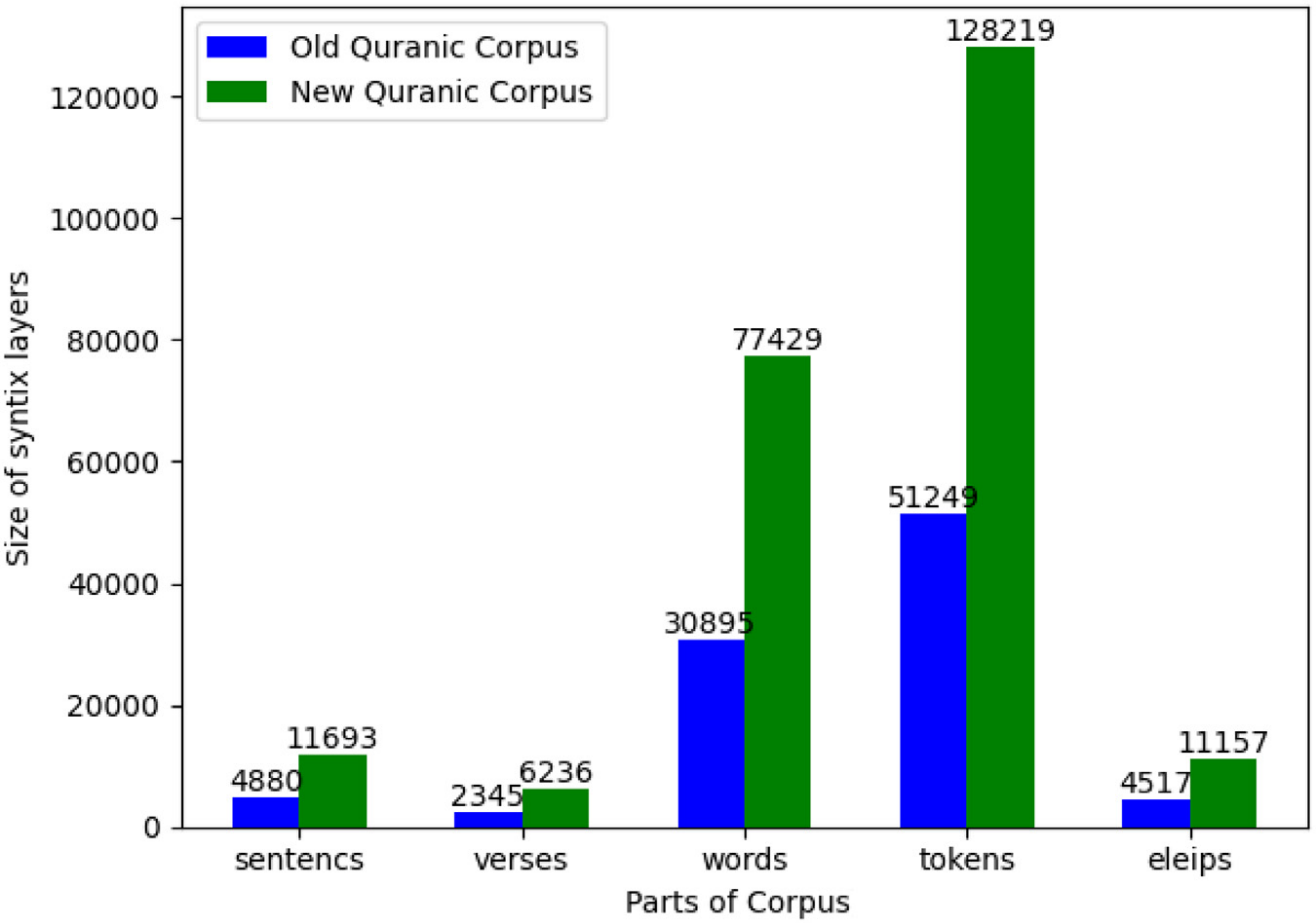
Size of the dataset before and after expansion.

As highlighted by this comparison, this substantial expansion—particularly the introduction of a complete and computationally accessible syntactic layer—dramatically elevates the dataset's scale and analytical capacity. Quantitative metrics, illustrated in Fig. 3, demonstrate significant growth; the dataset now encompasses fully parsed syntactic structures for over 11,693 sentences and 77,429 words (representing approximately a 1.5-fold increase over the effectively processable units in the previous resource). Furthermore, the annotation now explicitly captures elements like elliptical constructions, with 128,219 related instances identified.

**Fig. 4:**
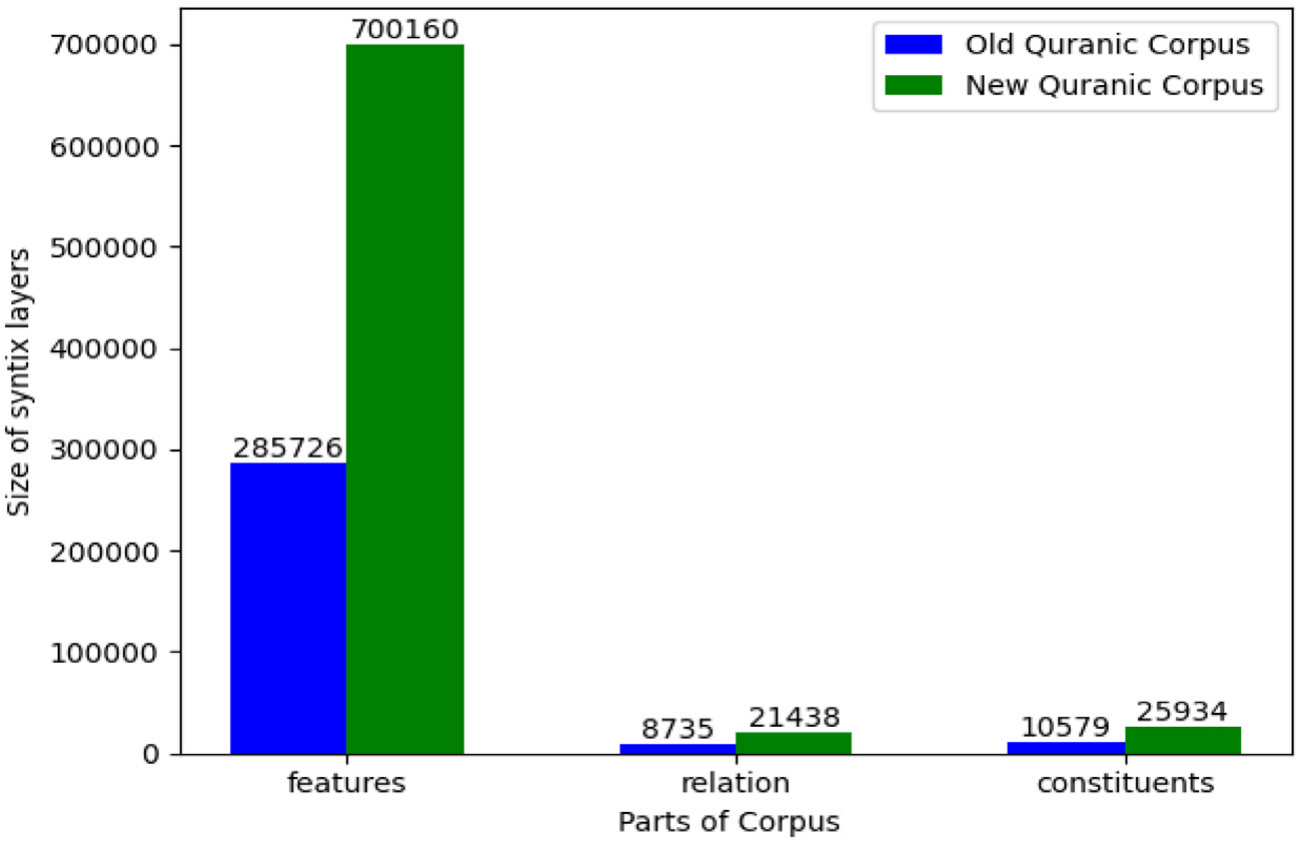
Impact of Syntactic Layer Expansion on the Structure of the Dataset.

The enhanced structural depth provided by the syntactic layer is further evidenced (Fig. 4) by a more than doubling of captured binary syntactic relations (over 21,400) and annotated morphological features (over 700,000), alongside a 1.5-fold increase in identified embedded phrasal constituents (nearly 26,000). Critically, this comprehensive syntactic information is delivered entirely in a machine-readable text format, overcoming the severe limitations imposed by the previous resource's reliance on non-processable, image-based representations for its partial syntactic coverage.

Ultimately, the provision of this fine-grained, expansive, and computationally tractable syntactic data for the entire Quran unlocks unprecedented opportunities. It furnishes a robust foundation for empirical research into the intricacies of CA sentence structure, facilitates the development and evaluation of sophisticated CA parsers (dependency, constituency, or hybrid), and enables the creation of advanced, syntax-aware language learning technologies, particularly beneficial for non-native speakers.

## 5 Experimental Design, Materials and Methods

The generation of this comprehensive CA Treebank involved a structured, multi-phase process focused on the systematic expansion, enhancement, and completion of annotations across three distinct linguistic layers: orthographic, morphological, and syntactic. The methodology leveraged existing foundational resources, primarily the Tanzil project dataset [1] and the Quranic Corpus project [2], coupled with the development and application of custom algorithms, rule-based systems, and machine learning models. The authors facilitated the subsequent manual validation by developing the review systems, training the participants, and providing continuous technical support. This validation was conducted by a dedicated team of volunteers, organized into two groups: a panel of six trained annotators, who were distinguished undergraduate students from a Quranic studies program selected for their advanced proficiency in Classical Arabic, and an expert panel of three senior reviewers, who were volunteer academics holding PhDs in Arabic Language and Linguistics. The expert panel provided oversight and adjudicated complex cases, with the specific involvement of these groups varying across the different linguistic layers as detailed below.

### 5.1 Orthographic layer extending

**Fig. 5:**
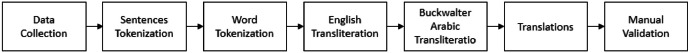
The seven-step process utilized to construct the Orthographical data.

The construction of the enriched orthographic layer followed a structured seven-step workflow (Fig. 5), designed to ensure both linguistic accuracy and computational interoperability. The process began with data collection, where the Imlaai script was sourced from the Tanzil project [1], while the Uthmani script and preliminary positional and morphological data were retrieved from the Quranic Corpus [2]. These two resources served as the foundational textual base for all subsequent processing.

Tokenization was then carried out using custom-built algorithms at both the word and sentence levels. A primary design principle was to maintain strict alignment with the word and morpheme segmentation of the original QADB to ensure data integrity and prevent segmentation mismatches. For word-level segmentation, which involves decomposing a single Quranic word (e.g., “”) into its constituent morphemes (tokens: ), our process was anchored to the QADB's segmentation scheme. To achieve this, we developed a lexicon-based algorithm that systematically replicates the QADB's morpheme boundaries. A critical component of this process was the construction of a rule-based, semi-automated character-level alignment map to reconcile orthographic differences between the Imlaai and Uthmani scripts. The map's primary function was to project the established morpheme boundaries from the Uthmani-based QADB onto the corresponding Imlaai text. The procedure was as follows: for tokens with identical spellings, boundaries were transferred directly. However, for instances with orthographic variations—such as the representation of a long vowel 'ā' as a dagger alif in Uthmani versus a full alif in Imlaai—a set of deterministic, script-to-script transformation rules was applied. To ensure accuracy, the validity of each resulting morpheme in the Imlaai script was then cross-checked against a purpose-built electronic lexicon derived from Khadir’s work [13]. This two-pronged approach (rule-based mapping and lexical validation) guaranteed that morpheme-level consistency was preserved across both script variants for every word in the corpus.

Sentence-level tokenization presented a significant challenge, given the absence of modern punctuation and the complex relationship between verses and grammatical sentences. We adopted a synergistic hybrid methodology, beginning with an automated first pass where heuristic rules generated preliminary candidate boundaries. The definitive segmentation was then established through a meticulous manual protocol where each of the three senior experts first independently segmented the entire corpus. Subsequently, these three parallel versions were collated, and a final, unified segmentation was determined through the structured adjudication protocol detailed in the validation process below. This methodology ensures a robust and linguistically principled result. It is important to note that this foundational sentence structure is further refined during the syntactic annotation phase, where tokens for elliptical elements are inserted and a final re-indexing is performed, as detailed in the Syntactic Layer Building section.

Following the tokenization stage, a sequence of data enrichment steps was executed to enhance the dataset's computational and pedagogical utility. First, to ensure compatibility with standard Arabic NLP tools, a Buckwalter transliteration for the Imlaai script was generated. This was achieved using a custom rule-based algorithm designed to systematically map Imlaai characters to their Buckwalter equivalents, resolving orthographic differences from the Uthmani script (such as the dagger alif). This newly generated transliteration complements the existing Uthmani Buckwalter data sourced from the Quranic Corpus [2]. Subsequently, a detailed English phonetic transliteration was produced for each word to support pedagogical applications and phonological analysis. Finally, English translations sourced from the Tanzil and Quranic Corpus websites [1,2] were integrated; a specialized matching algorithm was employed to contextually distribute the verse-level translations across their constituent words, thereby preserving alignment and integrity at both the verse and word levels.

The process concluded with a rigorous validation phase for both word- and sentence-level tokenization, conducted by the three-member senior expert panel. For word segmentation, the process began with independent reviews, which revealed a high degree of initial alignment, as reflected by a Fleiss’ Kappa score of 98.6 %. The few remaining disagreements were resolved through collective deliberation to achieve unanimous consensus on every morpheme boundary, establishing a highly reliable baseline for word tokenization. For the more interpretively complex task of sentence segmentation, a structured, multi-tiered adjudication protocol was employed. Independent reviews yielded a Fleiss’ Kappa score of 82.4 %, indicating strong agreement, and decisions were finalized based on majority consensus. In the rare instances where no majority was reached, the issue was escalated for final arbitration to a pre-designated senior linguist acting as a tie-breaker. While this comprehensive validation protocol ensures a robust and principled segmentation for the entire corpus, the project acknowledges that establishing a definitive 'gold standard' for sentence boundaries in classical texts is an iterative process. This layer, therefore, provides a strong and systematically validated foundation suitable for immediate computational use and as a basis for future refinement studies.

### 5.2 Morphological layer enhancement

**Fig. 6:**
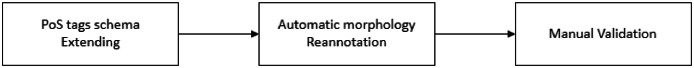
The three-step process utilized to prepare the dataset in the morphological layer.

The enhancement of the morphological layer was achieved through a meticulously structured, three-stage process designed to maximize both linguistic depth and computational utility. The initial phase focused on extending and refining the morphology annotation scheme. Building upon the foundational schema of the original Quranic Corpus, this stage began with a comprehensive review of the seven authoritative references in classical Arabic grammar and Quranic analysis [[14], [15], [16], [17], [18], [19], [20]]. This review confirmed that the existing Part-of-Speech (POS) tagset was sufficiently comprehensive and required no expansion. In contrast, the inventory of morphological features was found to be limited. Consequently, a systematic extraction of all morphological concepts from the references was conducted, yielding an extensive initial list of potential features. This list was then subjected to a rigorous consolidation phase overseen by an expert panel, which filtered it based on two primary criteria: frequency of attestation across the references and fundamental grammatical importance for syntactic analysis. Redundant or overlapping features were merged, while those deemed less critical or computationally intractable were discarded, resulting in the final, fine-grained schema of 20 distinct features Fig. 6.

With the extended schema in place, the process advanced to a dual-pronged automatic annotation phase. For Part-of-Speech (POS) tags, where the tagset remained unchanged, the focus was on validating the accuracy of annotations from the original QADB. A custom algorithm was developed to systematically compare each existing annotation against the analyses in the seven classical references [[14], [15], [16], [17], [18], [19], [20]], flagging only the discrepancies for subsequent manual review. Concurrently, a second, more generative algorithm was engineered to populate the 20 newly established morphological feature columns. This algorithm was designed to infer and assign feature values for every token by interpreting the traditional grammatical explanations found within the source texts, thus systematically creating a rich layer of data that was previously absent.

The final stage of the process was a comprehensive validation protocol designed to ensure the linguistic integrity of the enhanced morphological layer. The workflow mirrored the team-based structure used for syntactic validation: six trained annotators, specifically prepared for this task through targeted instruction in morphological annotation, conducted the primary layer of review, while the three senior experts provided ultimate oversight and adjudicated all complex or ambiguous cases. The training process was relatively straightforward, as the terminology used in the annotation schema was largely derived from traditional Classical Arabic grammar, making it familiar and intuitive for the annotators. To streamline this process, annotators utilized a custom-built interface that facilitated direct comparison between the dataset's annotations and the analyses presented in seven authoritative grammatical references [[14], [15], [16], [17], [18], [19], [20]].

The validation methodology itself was critically differentiated based on the nature of the annotation type. For the flagged POS tag discrepancies, a strict, multi-tiered protocol was enforced, requiring an annotation to be explicitly attested in at least four of the seven references [[14], [15], [16], [17], [18], [19], [20]] (“presence threshold”) and match at least 75 % of those sources (“agreement threshold”). In contrast, a more inclusive protocol was adopted for the newly generated morphological features, where a feature value was accepted for review even if attested in just a single reference—a policy acknowledging that such details are often considered implicit or are contextually understood in classical grammatical works.

In all cases where annotations failed to meet their respective thresholds or where references presented conflicting interpretations, the issue was escalated to the expert panel for final adjudication, with decisions settled by a majority vote. To further support these expert decisions and assess the level of inter-annotator agreement in ambiguous or underspecified cases, Fleiss’ Kappa was computed for a subset of morphological features subjected to manual arbitration. The results showed varying levels of agreement depending on the morphological clarity and linguistic complexity of each feature. Notably, the “gender” and “number” columns exhibited very high agreement, with Kappa scores of approximately 0.96 and 0.94, respectively. These features are morphologically explicit in Classical Arabic and are typically marked with unambiguous surface forms. In contrast, features such as “derived_nouns” and “verb_voice” yielded lower agreement scores, around 0.82 and 0.78, respectively. This reflects the greater interpretive demands of derived forms and internal morphological processes. Based on the evaluated subset, the overall mean Fleiss’ Kappa was estimated at 0.87, indicating substantial agreement and supporting the reliability of expert adjudications in the more challenging cases.

To further bolster consistency and support the experts' work, rule-based inference algorithms were also developed for features governed by deterministic grammatical rules, providing a reliable baseline for resolving complex cases. This hybrid approach—combining targeted algorithmic processing, differentiated validation protocols, and expert arbitration—produces a morphological layer that is both empirically robust and theoretically sound. While resource constraints did not permit an exhaustive, token-by-token double-annotation process for establishing a formal 'gold standard,' this systematic protocol guarantees that the annotations are of high quality, deeply rooted in traditional grammatical analysis, and ready for advanced computational and linguistic research.

### 5.3 Syntactic layer building

**Fig. 7:**
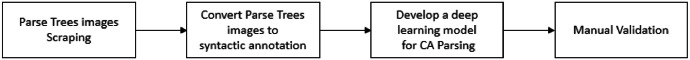
The Methodology of Syntactic Layer Building.

The construction of the syntactic annotation layer was guided by a comprehensive methodology—illustrated in Fig. 7—designed to ensure both full textual coverage and high linguistic accuracy. The process began with the acquisition of syntactic tree images from the Quranic Corpus website [2], which provided a foundational seed dataset covering approximately 40 % of the Quranic verses. While these tree structures conveyed human-readable syntactic information, they were only available as static images and thus required extensive transformation to become computationally tractable.

To extract structured syntactic data from the static tree images, a multi-stage conversion pipeline was developed, combining Optical Character Recognition (OCR) using the Tesseract engine—tuned for Arabic script—with geometric image processing techniques such as Hough Line Transform and contour detection. These methods enabled the recovery of node content and the inference of spatial relationships, which were then modeled as directed graphs and traversed recursively to reconstruct preliminary parse trees. However, the automated output exhibited notable inconsistencies due to OCR errors and visual complexity in the source images, making full automation impractical. To address these challenges, a dedicated visual alignment interface was implemented, allowing side-by-side comparison between each syntactic tree image and its corresponding parsed output. The manual correction was systematically carried out by the team of six trained annotators following a structured, dual-pass protocol. Initially, the complete set of generated trees was divided among the annotators, with each responsible for a primary review and correction of their assigned portion. To ensure robustness and inter-annotator consistency, a second pass was conducted where the corrected portions were exchanged among the annotators for a cross-review. This protocol ensured that every generated tree was independently validated by two different annotators. While the conversion process required significant human intervention, this dual-pass correction protocol ensured a high level of structural fidelity, resulting in a curated dataset suitable for training and evaluating syntactic parsers with confidence.

Fundamental to the parsing and validation stages was the development of a comprehensive, hybrid annotation scheme capable of capturing the complexities of CA syntax. The design process was grounded in the same seven authoritative grammatical references [[14], [15], [16], [17], [18], [19], [20]]. Following an exhaustive inventory of possible dependency relations compiled from these sources, the expert panel conducted a multi-stage consolidation process. This involved merging synonymous labels from different references, grouping related sub-types, and standardizing terminology. Final decisions on the inclusion and definition of each label required a majority consensus among the panel members, a protocol that ultimately resulted in the final, fine-grained dependency tagset of 140 distinct relation labels (summarized in the RelLabels file). In contrast, for phrasal representation, the panel reviewed the existing set of 7 constituent tags from the original corpus, confirmed their suitability, and consequently retained them within the new scheme (as defined in the ConstituentsTags file).

Before parsing, a critical pre-processing stage was performed to create a syntactically complete and computationally optimized version of the corpus. A dedicated module, named TAQDIR, was employed to systematically detect and resolve elliptical constructions. This module operates through a hybrid methodology, integrating a rule-based component grounded in classical Arabic grammar with context-aware heuristics. The rule-based system addresses high-confidence, predictable scenarios by identifying obligatory but unfilled syntactic slots—for example, a nominal sentence topic (mubtadaʾ) that lacks an explicit predicate (khabar). The heuristic component complements this by addressing more ambiguous contexts through pattern matching against canonical sentence structures. Based on the output of this process, the module inserts linguistically motivated placeholders for non-overt elements; for instance, an omitted noun or verb is represented by a generic placeholder token (*), while an omitted pronoun is inserted explicitly (e.g., ). Crucially, each inserted placeholder is also assigned a Part-of-Speech (POS) tag (e.g., NOUN, PRON) to provide the parser with essential grammatical context.

The integration of these elliptical tokens led to a foundational design decision: to establish a new, improved standard for the corpus structure. Following the insertion process, a complete re-indexing of all tokens (both original and inserted) was performed on a sentence-by-sentence basis. This “new standard” approach creates a simple, contiguous, and sequential index (e.g., 1, 2, 3, …N) for each sentence. While this method intentionally breaks backward compatibility with the original QADB's location-based structure, it provides a structurally coherent and computationally clean representation, drastically simplifying the implementation of parsing algorithms. To ensure continued interoperability, each token entry in the updated corpus retains its original QADB location identifier alongside the newly assigned sequential token index, allowing for direct alignment and traceability.

With this processed and re-indexed data as a foundation, a deep learning-based syntactic parser was developed to generate the annotations. The parser's architecture was centered on a shared Bidirectional Long Short-Term Memory (BiLSTM) encoder composed of two layers, with a standard dropout of 0.3 and a recurrent dropout of 0.1 applied to prevent overfitting. For the input layer, each token was represented by a concatenated vector of three distinct embeddings: (1) 300-dimension pre-trained Word2Vec embeddings derived from a large corpus of classical Arabic texts from the Comprehensive Islamic Library [3], (2) 50-dimension Part-of-Speech (POS) tag embeddings, and (3) 100-dimension embeddings representing the token's fine-grained morphological features.

The context-rich representations generated by the BiLSTM encoder served as input for two parallel output modules, each employing a specialized transition-based system. The dependency parsing module utilized the ARC-IIRAB system, which was specifically engineered to manage complex characteristics of CA syntax, such as non-projective structures and subject ellipsis. In parallel, the constituency parsing module employed the ARC-MAHAL system, a lightweight framework designed to identify only the most functionally significant phrasal units (e.g., mubtadaʾ, khabar) in line with traditional Arabic grammar, rather than generating an exhaustive phrase-structure tree.

The model was implemented using the TensorFlow v2.8 framework and trained on a reserved portion of the structured seed data. The training process utilized the Adam optimizer with a learning rate of 0.001, a batch size of 64, and was regulated by an early stopping protocol (patience=5) and a ReduceLROnPlateau learning rate scheduler (factor=0.5, patience=2). Once optimized, the parser was applied to the entire corpus to generate an initial, complete hybrid syntactic layer integrating both dependency and constituency information. Recognizing the inherent limitations of automated parsing for a language as complex as Classical Arabic, this automatically generated output was not considered final, but rather served as the foundational draft for the systematic manual validation phase that followed.

Recognizing the inherent limitations of automated parsing for a language as complex as Classical Arabic, the parser's output was not considered final. Instead, it served as a foundational draft for a systematic validation and refinement phase led by the full annotation and review team. The six trained annotators, who had previously participated in the morphological annotation phase, relied on that experience as a starting point for engaging with the syntactic layer. However, this layer presented greater conceptual and structural complexity. While core dependency relations—such as fāʿil (subject), mafʿūl bihi (object), and muḍāf ilayh (genitive complement)—were largely intuitive, several of the newly introduced fields posed notable challenges. In particular, identifying syntactic heads (ref_token_id), determining whether a token served as a constituent root (is_constituent), and specifying the span of each constituent (constituents_loc) often required abstract reasoning and fine-grained interpretation—especially in the presence of ellipsis, coordination, or embedded structures. These complexities necessitated targeted clarification during training and contributed to a steeper learning curve compared to earlier annotation phases.

To manage this complexity, the annotators carried out the validation work themselves, with direct supervision from a panel of three senior experts in computational linguistics and Classical Arabic grammar (iʿrāb). The role of this expert panel was primarily advisory—providing methodological guidance, resolving annotation conflicts when they arose, and overseeing the integrity of the process. To support consistency and traceability, a custom-built annotation interface was employed, enabling annotators to compare syntactic parses against analyses from seven authoritative reference works [[14], [15], [16], [17], [18], [19], [20]].

The annotation process was governed by a rule-based protocol intended to maximize objectivity. Under this protocol, a syntactic annotation was accepted only if it satisfied two criteria: (1) an existence threshold—explicitly attested in at least four of the seven reference works [[14], [15], [16], [17], [18], [19], [20]]; and (2) an agreement threshold—with at least 75 % alignment among the attesting sources. In principle, cases failing to meet these criteria or showing conflicting interpretations were to be escalated to the expert panel for final arbitration by majority vote. However, due to limited project support, many such edge cases could not be formally reviewed by the panel and were instead adjudicated by annotators based on the best alignment with the reference sources. This practical compromise ensured broad adherence to the protocol while maintaining forward progress.

Importantly, while the expert committee supervised the overall process, they did not engage in full double-annotation or systematic adjudication of all ambiguous cases. As a result, formal inter-annotator agreement (IAA) metrics were not computed across the entire corpus. Instead, a representative sample of 350 sentences was selected and evaluated using two widely recognized metrics: the Labeled Attachment Score (LAS) for dependency parsing, which achieved 95.7 %, and the PARSEVAL F1-score for constituent structures, which reached 93.3 %. These results indicate strong internal consistency and affirm the reliability of the annotated syntactic layer despite the project’s resource constraints.

To support the practical use of the annotated treebank, an advanced visualization and analysis platform was developed and is publicly accessible at https://github.com/NoorBayan/Noor. This interface enables dynamic exploration of the morphological and syntactic layers, and it is explicitly designed to resonate with traditional Arabic grammar concepts. A key feature of this tool is its graphical rendering capability, hosted at https://github.com/NoorBayan/Quranic, which uses Scalable Vector Graphics (SVG) to represent syntactic structures in a visually intuitive manner. Each node displays core morphological information (e.g., POS, case, number), and syntactic dependencies are visualized as labeled arcs. The structure of each tree also reflects traditional grammatical relations such as āmil (governing element) and maḥall (grammatical slot), providing a bridge between computational syntax and classical linguistic analysis. SVG was chosen for its high rendering precision, scalability, and ease of integration into digital humanities platforms, making it suitable for both scholarly research and educational purposes.

## Limitations

While this dataset provides a comprehensive, multi-layered resource for CA, including a novel, complete syntactic layer for the entire Quranic text, a primary limitation pertains to the current validation status of this newly generated syntactic annotation. The construction methodology involved automated parsing followed by a validation phase guided by grammatical rules formulated by linguistic experts. However, the syntactic layer has not yet undergone the exhaustive, token-by-token manual review and inter-annotator agreement adjudication typically associated with establishing a definitive “gold standard” corpus. Consequently, users leveraging this dataset, particularly for tasks heavily reliant on the precision of the syntactic annotations, should be cognizant that residual errors or inconsistencies, inherent to automated parsing followed by rule-based validation rather than full manual curation, may be present. This limitation primarily affects the syntactic layer; the orthographic and morphological layers benefited from different construction and refinement processes.

## Ethics Statement

The authors have read and follow the ethical requirements for publication in Data in Brief and confirming that the current work does not involve human subjects, animal experiments, or any data collected from social media platforms.

## CRediT Author Statement

**Wadee A. Nashir:** Conceptualization, Resources, Methodology, Data curation, Visualization, Software. **Abdulqader M. Mohsen:** Supervision, Writing – original draft preparation, Writing – review & editing. **Asma A. Al-Shargabi**: Validation, Visualization, Investigation, Writing – review & editing. **Mohamed K. Nour:** Conceptualization, Writing – review & editing. **Badriyya B. Al-onazi:** Conceptualization, Validation, Visualization.

## Data Availability

Mendeley DataQuranic (Original data).
